# Regulation of L-type Voltage Gated Calcium Channel CACNA1S in Macrophages upon *Mycobacterium tuberculosis* Infection

**DOI:** 10.1371/journal.pone.0124263

**Published:** 2015-04-27

**Authors:** Cecil Antony, Subhash Mehto, Brijendra K. Tiwari, Yogendra Singh, Krishnamurthy Natarajan

**Affiliations:** 1 Infectious Disease Immunology Lab, Dr. B. R. Ambedkar Centre for Biomedical Research, University of Delhi, Delhi, 110007, India; 2 CSIR-Institute of Genomics and Integrative Biology, Mall Road, Delhi, 110007, India; Public Health Research Institute at RBHS, UNITED STATES

## Abstract

We demonstrated earlier the inhibitory role played by Voltage Gated Calcium Channels (VGCCs) in regulating *Mycobacterium tuberculosis (M*. *tb)* survival and pathogenesis. In this report, we investigated mechanisms and key players that regulate the surface expression of VGCC-CACNA1S by Rv2463 and *M*. *tb* infection in macrophages. Our earlier work identified Rv2463 to be expressed at early times post infection in macrophages that induced suppressor responses to dendritic cells and macrophages. Our results in this study demonstrate a role of MyD88 independent TLR pathway in mediating CACNA1S expression. Dissecting the role for second messengers, we show that calcium homeostasis plays a key role in CACNA1S expression during *M*. *tb* infection. Using siRNAs against molecular sensors of calcium regulation, we show an involvement of ER associated Stromal Interaction Molecules 1 and 2 (STIM1 and STIM2), and transcription factor pCREB, towards CACNA1S expression that also involved the MyD88 independent pathway. Interestingly, reactive oxygen species played a negative role in *M*. *tb* mediated CACNA1S expression. Further, a cross-regulation of ROS and pCREB was noted that governed CACNA1S expression. Characterizing the mechanisms governing CACNA1S expression would improve our understanding of the regulation of VGCC expression and its role in *M*. *tb* pathogenesis during *M*. *tb* infection.

## Introduction

Tuberculosis, caused by its etiological agent *Mycobacterium tuberculosis* (*M*. *tb*), remains a major killer till date [1]. 2012 estimates of WHO put the figures of people infected with *M*. *tb* at 8.3 million new cases with 1.3 million deaths annually [2]. *M*. *tb* employs multiple mechanisms to evade immune responses. The pathogen remains undetected within the host by modulating pathways that are responsible for recognition and elimination of the pathogen [3–5]. *M*. *tb* prevents phagolysosome fusion [6] and remains undetected within phagosomes, inhibits apoptosis, autophagy and downregulates the surface expression of Interferon gamma receptor [7–9]. A key molecule that regulates many of these processes during infections is calcium [10, 11]. Calcium plays a definite role in regulating the pathogenesis of *M*. *tb* [10–12] that include activation of transcription factors, mediating phagolysosome fusion, cell survival etc [13–15]. Calcium fluxes in response to various stimuli govern the selective activation and inactivation of transcription factors resulting in altered genotypic and phenotypic outcomes [16]. Calcium influx facilitates the activaiton of CREB that in turn initiate a multitude of cellular processes. A key process that is regulated by CREB in macrophages is the activation of anti-apoptotic pathway. A number of pathogens such as *Shigella*, *Salmonella* and *Yersinia* utilize this mechanism to activate CREB via calcium influx and suppress protective immune responses [17–18]. Similary, the lethal toxin of *Bacillus anthracis* also activates CREB to inhibit macrophage apoptosis [19]. Furthermore, CREB induced TNF-alpha production promoted anti-apoptotic responses in macrophages [20].

Calcium influx in cells is primarily in two phases [21]. In the first phase there is a depletion of intracellular stores from the ER that opens up specific channels in the plasma membrane. The second phase is called the capacitative phase and leads to a sustained increase in intracellular calcium concentrations [22]. This second phase of calcium influx is either via calcium release activated calcium channels (CRACs) or via Voltage Gated Calcium Channels (VGCCs) or both [23]. The role of VGCCs has been extensively studied in physiological and neurological conditions [24, 25] and its role in pathological infections is now fast emerging [26–28]. Further, both molecular genetic and pharmacological approaches have revealed the existence of functional L-type VGCCs in a variety of hematopoietic cells such as dendritic cells [29], neutrophils [30], NK cells [31], T cells [32–35] and B cells [36].

Our work in this direction has identified important roles for VGCCs in regulating *M*. *tb* pathogenesis [37]. Inhibiting VGCCs negatively regulated host immune responses by affecting cytokine and chemokine profiles and modulated genes involved in antigen presentation and T cell priming. Blocking L-type VGCC increased calcium influx from CRAC channels in infected cells. Peripheral Blood Mononuclear Cells (PBMCs) of tuberculosis patients expressed higher levels of these channels when compared to healthy controls which were reduced following chemotherapy [37]. Similar observations were later made by two independent groups [38–39]

Therefore, in this study we investigated the regulation of CACNA1S expression in murine macrophages in the context of *M*. *tb* antigenic stimulation and live infection. Several studies document the similar responses of human and mouse macrophages to mycobacteria. For example, IL-6 produced from *M*. *tb* infected macrophages selectively inhibits IFN-γ responses in both human and murine macrophages [40]. Enhanced superoxide burst and reduction in lipolytic activity of phagosomes post infection were also reported to be similar in both human and mouse [41]. We chose to work with Rv2463, an antigen that we identified in our earlier study [42]. Stimulation of dendritic cells (DCs) and macrophages with Rv2463 mediated suppressor responses including downregulation of IL-12p40 from dendritic cells and induction of Th2 responses from T cells. Further, overexpression of this antigen in DCs increased intracellular bacterial burden. Since we also demonstrated the inhibitory role of VGCC [37], we hypothesized that Rv2463 could regulate the expression of CACNA1S, a calcium channel whose expression and regulation has been detected in T cell subsets but not in macrophages, especially in the context of *M*. *tb* infection. It was shown that reduced expression levels of CACNA1S leads to decreased production of Interleukin 2 (IL-2), less proliferation of CD4^+^ T cells, defective cytotoxic T lymphocyte (CTL) responses and therefore hampers Ca^2+^ signaling [28, 43–45]. However, there are no reports on the role of calcium homeostasis, oxidative burst and molecules that regulate calcium influx like Stromal Interaction Molecule (STIM), and key transcription factors in regulating VGCC expression. Therefore, in this report, we investigated the ability of Rv2463 and *M*. *tb* live infections in regulating CACNA1S expression and the role of innate receptors and downstream signaling molecules thereof. Our results point to a role of Myeloid Differentiation primary response 88 (MyD88) independent Toll Like Receptor (TLR) pathway, calcium homeostasis, the oxidative burst and transcription factor CREB in mediating CACNA1S expression on macrophages.

## Materials and Methods

### Materials

Antibodies to pCREB-1, SOX5, GATA2, specific and control siRNAs to various genes and luminol kits for chemiluminescence detection were purchased from Santa-Cruz Biotechnologies (Santa Cruz, CA). TMB8, EGTA and DPI were purchased from Sigma Chemical Co. (St. Louis MA). Recombinant Rv2463 was heterologously expressed in *E*. *coli* and affinity purified as described earlier [42, 46]. *M*. *tb* strain H37Rv was cultured in 7H9 broth (DIFCO) supplemented with 10% OADC (BD) and 0.05% Tween 80 and resuspended in 1X PBS at 1× 10^8^ CFU/ml.

### Animals

For some experiments, female 6–8 week old Balb/c mice kept in pathogen free environment were used. The experiments with animals had the prior approval of the insitutional animal ethics committee of Dr. B. R. Ambedker Center for Biomedical Research, University of Delhi.

### Stimulations and infection

Mouse macrophage cell line J774 was used throughout in the study. The cells were cultured in RPMI 1640 medium along with 10% Fetal Bovine Serum and antibiotics at 37°C, 5% CO_2_. Cells were either stimulated with 25 μg/ml of Rv2463 or infected with 2 Multiplicity of Infections (MOI) of *M*. *tb* H37Rv for indicated times. The following bio-pharmacological inhibitors were used against various molecules. Inositol 1, 4, 5-trisphosphate receptor (IP_3_R,), [3,4,5-trimethoxybenzoic acid 8-(diethylamino)octyl ester] (TMB-8, 100μM); calcium influx, Ethyleneglycol tetraacetic acid (EGTA, 3mM); Reactive Oxygen Species (ROS) and Diphyneleneiodonium (DPI, 10μM). Unless mentioned otherwise, cells were incubated with the above reagents for 1h prior to stimulation with Rv2463 or infection with *M*. *tb*.

### Differentiation of mouse bone marrow derived macrophages

For some experiments bone marrow precursors from 6–8 week old Balb/c mice were enriched. MHC class-II^+^ cells, B and T lymphocytes were removed by two rounds of incubation with I-A^+^, CD54R and CD90- coupled microbeads, respectively, followed by Magnet assisted Cell Sorting (MACS) (Miltenyi Biotech, USA). Enriched precursors were cultured in RPMI 1640 medium supplemented with 40 ng/ml Macrophage-Colony Stimulating Factor (M-CSF) (R&D Systems, Minneapolis, MN), for 3 days to differentiate them into macrophages. Differentiated macrophages were then either stimulated with 25 μg/ml of Rv2463 or infected with 2 MOI of *M*. *tb* H37Rv for indicated times and analysed by flow cytometry as above.

### Antibody biotinylation and flow cytometry

Antibody to CACNA1S (Santa Cruz Biotechnologies # sc-8160) was biotinylated using NHS biotin as per standard protocols. Briefly, 2 μg of antibody to CACNA1S was mixed in 0.1 M sodium carbonate buffer (NaHCO_3_/Na2CO_3_) pH 9.5 containing 0.1% NaN_3_. NHS-D-Biotin (Sigma) at a concentration of 22 mg/ml was added to 10% of the total volume of the immunoglobulin solution. After gentle mixing the contents were incubated at room temperature for 4 h. The reaction contents were dialyzed in PBS buffer (0.01 M sodium phosphate, 0.15 M sodium chloride, pH 7.4, containing 0.1% NaN_3_) at 4°C. For flow cytometry, cells were first incubated with Fc-block (BD Biosciences) followed by incubation with the above antibody at 1 μg/10^6^ cells at 4°C for 30 min. Cells were washed and counter-stained with streptavidin-FITC and for all experiments 10,000 events were acquired on FACSCalibur (BD BioSciences, USA). Further the data were analyzed using CellQuest Pro software. No gates were applied for analyses.

### Internalization of Rv2463 by macrophages

Rv2463 was biotinylated using NHS biotin as per standard protocols. J774 macrophages were stimulated with PE-streptavidin-biotin conjugated Rv2463 (25 μg/ml) for different time intervals (30 min). Z-stack images were collected at 1 μm intervals for internalization using confocal microscopy (Nikon C2).

### Confocal microscopy

At the end of incubation cells were fixed with acetone: methanol 1:1 for 20 min in 4°C. Cells were washed twice and incubated with antibodies against CACNA1S for 2h followed by addition of anti-rabbit FITC tagged Alexa flour 488 for 1.5h. Cells were again washed and mounted with anti-fade containing DAPI. Confocal imaging was performed on Nikon C2 laser scan confocal microscope (Nikon, Japan) with 60X objective magnification, numerical aperture 1.4, refractive index 1.5, Plan Apo optics equipped with Argon laser, using excitation and emission wavelength of 488 nm, respectively. Data were analyzed using the NIS Elements AR software.

### siRNA mediated gene knockdown

2 x 10^5^/ml J774 cells were transfected with 60 pmoles of siRNA against various genes for 36h using the Hiperfect transfection reagent (Qiagen) in OPTIMEM medium (Invitrogen) as described earlier [47]. Subsequently, cells were stimulated with Rv2463 or infected with 2 MOI of *M*. *tb* H37Rv for 24h or 48h and subjected to Fluorescence Activated Cell Sorter (FACS) or western blotting. The efficiency of knockdown is depicted in (S1 Fig).

### Western blotting for signaling molecules

Cytoplasmic and nuclear extracts were prepared as described earlier [48]. At the end of the incubation period, J774 cells were washed once with ice cold PBS and lysed in cytoplasmic extraction buffer (10 mM HEPES pH 7.9; 10 mM KCl; 0.1 mM EDTA; 0.1 mM EGTA, 0.5% Nonidet P-40, and 2 μg/ml each of aprotinin, leupeptin and pepstatin). The lysate was centrifuged at 13,000g for 1 min at 4°C. The nuclear pellet was resuspended in ice-cold extraction buffer (20 mM HEPES, pH 7.9; 0.4 M NaCl; 1 mM EDTA; 1 mM EGTA; 1 mM DTT; 1 mM PMSF and 2 μg/ml each of aprotinin, leupeptin and pepstatin) and incubated for 15min with brief vortexing intermittently to break open the nuclear membrane. The nuclear pellet was centrifuged at 13,000g for 5 min, at 4°C and the supernatant was designated as the nuclear extract. 25 μg micrograms of cytoplasmic extract or 10 μg of nuclear extracts was resolved on 12% denaturing SDS-PAGE followed by transfer onto nitrocellulose membrane (Hybond C pure, Amersham Biosciences). Membrane was blocked overnight with 5% BSA and later probed against molecules of interest with specific antibodies. After a brief wash with PBST (1X PBS, 0.05% Tween 20) HRP-labelled secondary antibodies were added as detection antibodies. The blots were later developed by chemiluminescence using the luminol reagent (Santa Cruz Biotechnology).

### Elecrophoretic Mobility Shift Assays (EMSA)

J774 macrophages were infected with 2 MOI *M*. *tb* or stimulated with 25 μg of Rv2463 for indicated times and nuclear extracts were prepared as described above. EMSA were performed by incubating 12–15 μg of nuclear extract with ^32^P-end-labeled 29-mer double stranded oligonucleotide sequence 5’-CAGATGGGGATGTCACCGGCTGGACTGGA-3’ and its complementary 5’-TCCAGTCCAGCCGGTGACATCCCCATCTG-3’ for 15 min at 37°C [49]. The incubation mixture included 2–3 μg of poly (dI.dC) in a binding buffer (25 mM Hepes, pH 7.9; 0.5 mM EDTA; 0.5 mM DTT; 1% Nonidet P-40; 5% glycerol; 50 mM NaCl). The DNA-protein complex formed was separated from free oligonucleotide on 5% native polyacrylamide gel using buffer containing 50 mM Tris, 200 mM glycine (pH 8.5), and 1 mM EDTA, and the gel was then dried. The specificity and extent of binding was examined by competition with unlabeled (cold) oligonucleotide.

### Measurement of Reactive Oxygen Species

Reactive Oxygen Species (ROS) levels were monitored as described earlier [46]. Briefly, 30min prior to the incubation period, cells were loaded with 10μM 2’, 7’-Dichloroflurescein diacetate (DCFH-DA). At the end of the incubation period, cells were thoroughly washed with culture medium. Cells were washed once again with culture medium and immediately analyzed for ROS levels by flow cytometry.

### Statistical analyses

Two-tailed Student’s t test was carried out to determine statistical significance. The P values are given at the end of the figure legends. P values < 0.05 were taken as statistically significant differences between compared groups.

## Results

### *M*. *tb* and Rv2463 upregulate CACNA1S expression in macrophages

**Fig 1:**
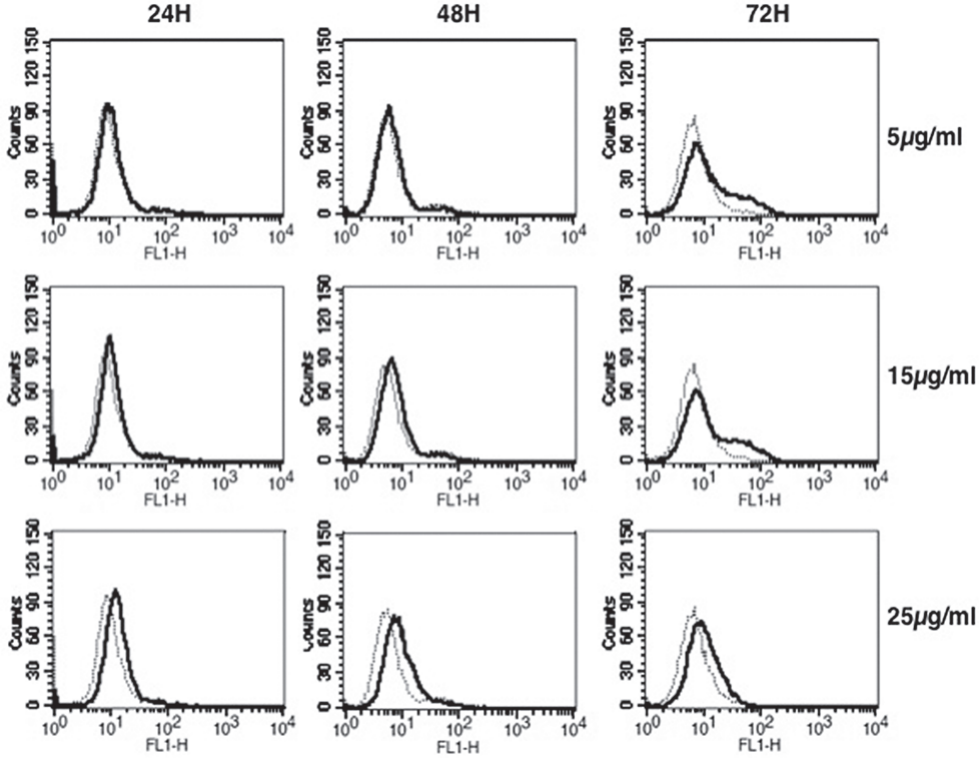
Rv2463 induces the upregulation of CACNA1S on macrophages. J774 macrophages were stimulated with Rv2463 at indicated concentrations and for indicated times. CACNA1S expression on cell surface was monitored by flow cytometry. Bold lines represent stimulations with Rv2463 while dotted lines represent unstimulated controls. Data from one of four independent experiments are shown. P<0.020; for unstimulated v/s 5 μg/ml of Rv2463; P<0.015, for unstimulated v/s 10 μg/ml of Rv2463; P<0.005, for unstimulated v/s 25 μg/ml of Rv2463. P values were calculated for 48h time point. Two-tailed Student’s t-test was employed for P values.

To begin with we investigated the ability of Rv2463 to modulate CACNA1S expression in mouse macrophages. To that end J774 cells were stimulated with different concentrations of Rv2463 for different time periods and surface expression of CACNA1S was monitored by flow cytometry. As shown in (Fig 1), Rv2463 increased the expression of CACNA1S in a dose and time dependent manner. Maximal effects were observed with 25 μg/ml of Rv2463 at 48h. We first ensured that J774 macrophages express basal levels of CACNA1S and also ensured the specificity of binding using an isotype matched control antibody (S2A and S2B Fig). We also investigated if the effects observed with Rv2463 are specific. To that end J774 macrophages were stimulated with another *M*. *tb* antigen, namely Rv3416. Rv3416 is another antigen identified by us and is expressed in infected macrophages at later times post-infection [42]. Like Rv2463, Rv3416 also induced suppressor responses to macrophages and DCs [42, 46]. However, unlike Rv2463, Rv3416 did not modulate CACNA1S expression on macrophages. No upregulation or downregulation of CACNA1S was observed in Rv3416 stimulated cells (S3 Fig). This indicated that the modulation of CACNA1S by Rv2463 was specific.

**Fig 2:**
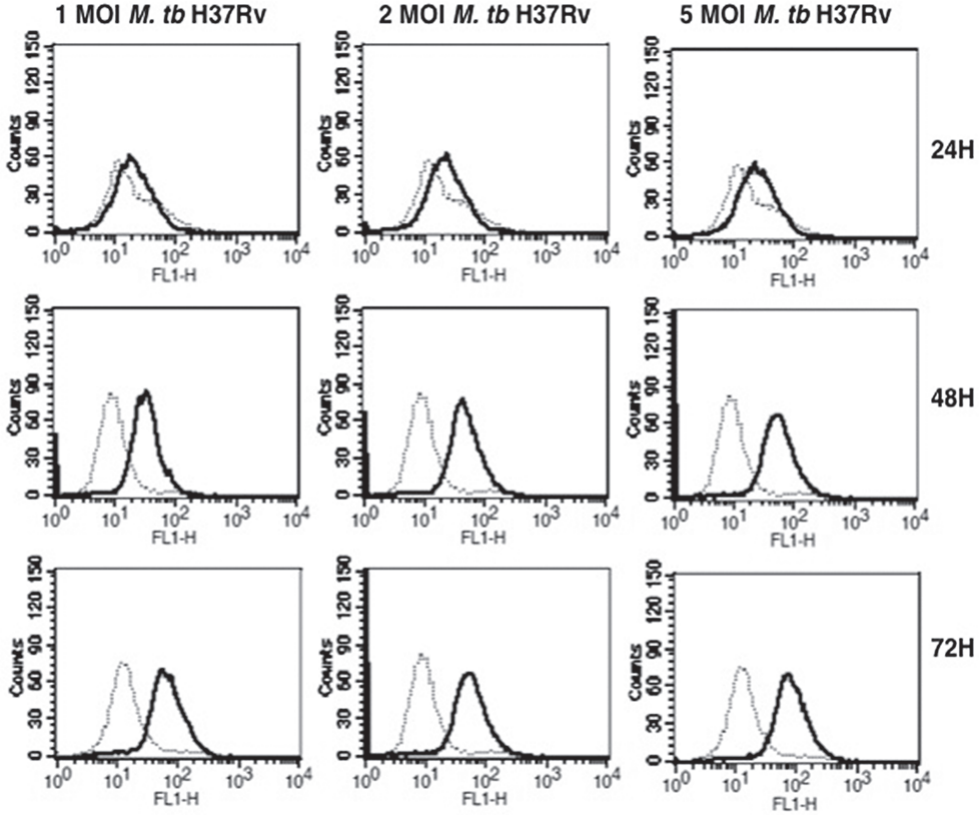
*M*. *tb* H37Rv induces the upregulation of CACNA1S on macrophages. J774 macrophages were infected with *M*. *tb* H37Rv at indicated Multiplicity of Infection (MOI) for indicated times. CACNA1S expression was monitored by flow cytometry. Bold lines represent infections with *M*. *tb* H37Rv while dotted lines represent uninfected controls. Data from one of four independent experiments are shown. P<0.011 for uninfected v/s 1 MOI of *M*. *tb* infected macrophages; P<0.004 for uninfected v/s 2 MOI of *M*. *tb* H37Rv infected macrophages; P<0.007 for uninfected v/s 5 MOI of *M*. *tb* infected macrophages. Two-tailed Student’s t-test was employed for P values at the 48h time point.

**Fig 3:**
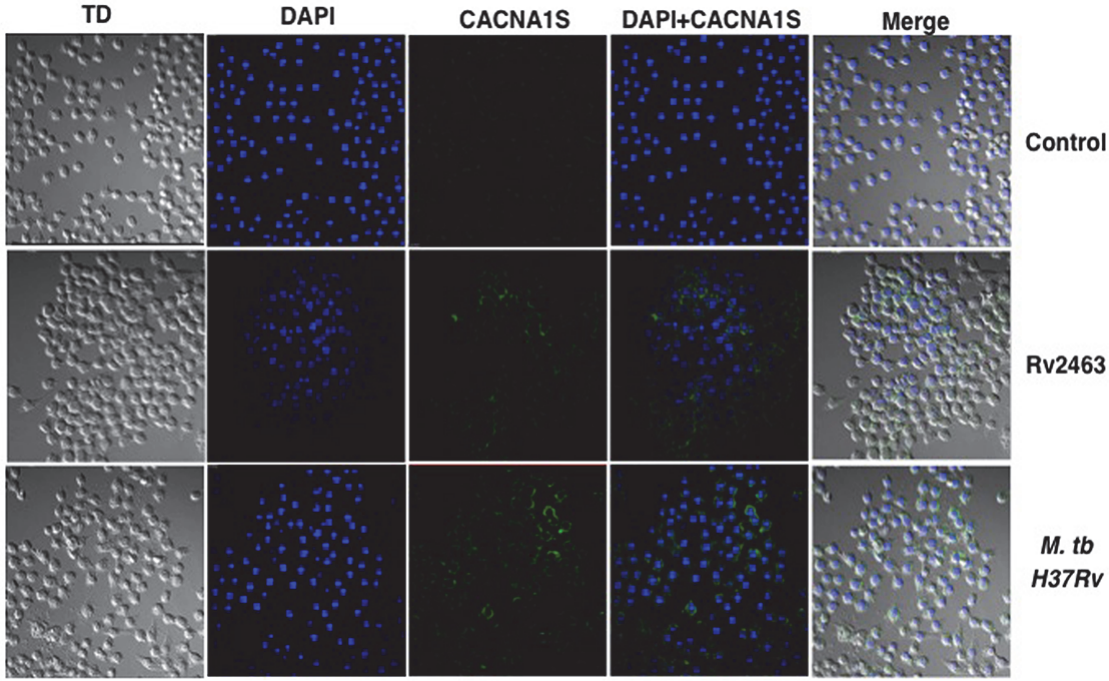
Confocal images of CACNA1S following stimulations with Rv2463 and *M*. *tb* on macrophages. J774 cells were stimulated with 25 μg/ml Rv2463 or 2 MOI *M*. *tb* H37Rv for 48h. CACNA1S expression was monitored by confocal imaging (*see Materials and Methods*). Blue colour represents staining of nucleus with DAPI while green colour represents CACNA1S staining with streptavidin conjugated FITC bound to biotinylated antibody to CACNA1S. A representative image of 10 fields is shown. P<0.009 for unstimulated v/s 25 μg/ml of Rv2463; P<0.004 for uninfected v/s 2 MOI of *M*. *tb* H37Rv infected macrophages. Two-tailed Student’s t-test was employed for P values for 48h time point.

To investigate if CACNA1S expression would be modulated by live *M*. *tb* infections, we infected macrophages with *M*. *tb* at different MOI as shown in (Fig 2), infecting cells with *M*. *tb* also increased CACNA1S expression in an MOI and time dependent manner. These results indicate that the effects observed with Rv2463 were reproducible with live infections. We also investigated the CACNA1S expression by confocal microscopy, as shown in (Fig 3), both Rv2463 and *M*. *tb* H37Rv increased CACNA1S expression on macrophage surface thereby confirming the data obtained by FACS. We further investigated whether Rv2463 acts from the cell surface or is taken up by macrophages. To that end J774 macrophages were stimulated with biotin labelled Rv2463 conjugated to streptavidin PE for 30 min and cells were analyzed by confocal microscopy. As seen in (S4 Fig), Rv2463 was completely internalized by macrophages within 30 min. This indicated that Rv2463 acted on macrophages following its internalization. However, at this moment the possibility of Rv2463 triggering an innate receptor immediately upon binding cannot be ruled out.

We further investigated if the responses observed with J774 cells could be replicated with primary cells. To that end bone marrow derived macrophages were either stimulated with Rv2463 or infected with *M*. *tb* H37Rv for 48h. As shown in (S5 Fig) and like J774, both Rv2463 as well as live *M*. *tb* infection upregulated the expression of CACNA1S. This indicated that the observed effects of Rv2463 or *M*. *tb* are not limited to macrophage cell lines.

### Rv2463 and *M*. *tb* regulate CACNA1S expression in a MyD88 independent pathway

**Fig 4:**
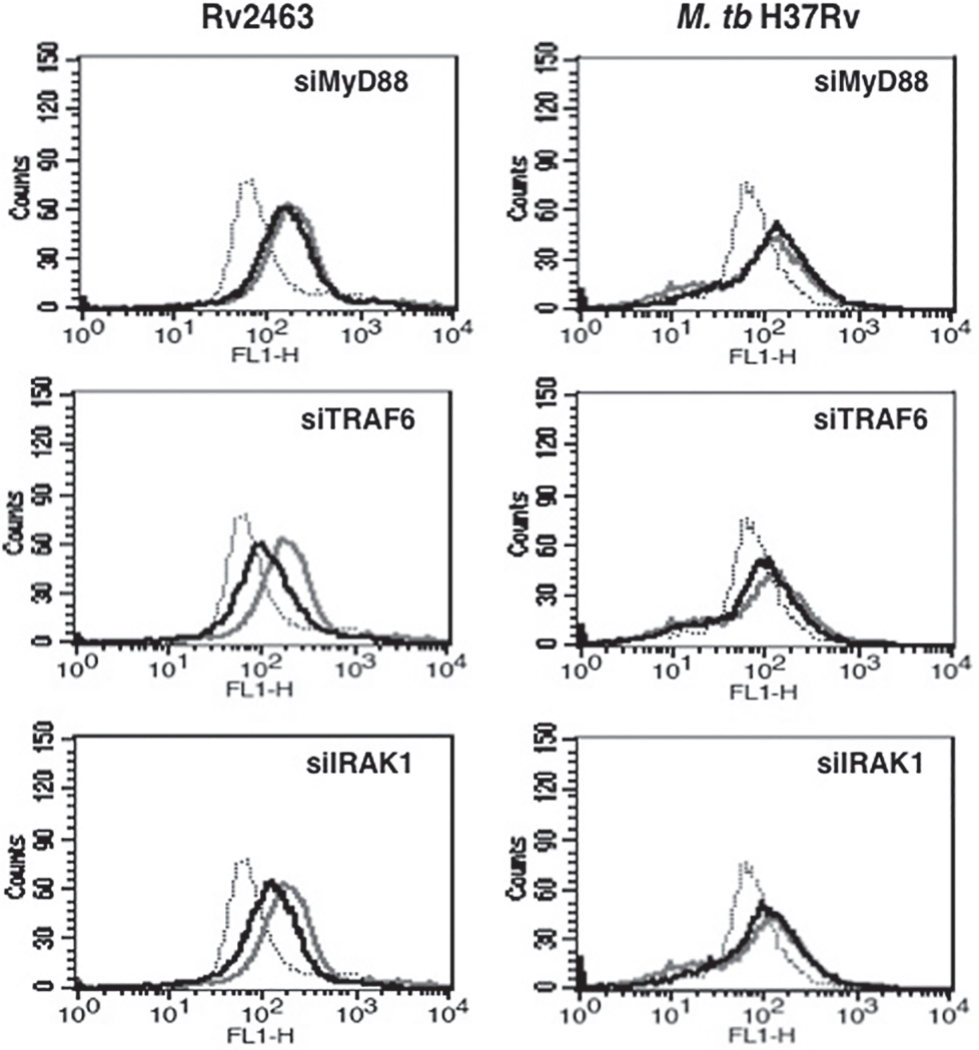
Rv2463 upregulates CACNA1S in a TLR dependent and MyD88 independent pathway on macrophages. J774 cells were transfected with siRNAs against MyD88, TRAF6 or IRAK1 for 36h followed by stimulations with 25 μg/ml Rv2463 or infection with 2 MOI of *M*. *tb* H37Rv for 48h. Surface CACNA1S expression was monitored by flow cytometry. Grey lines represent cells transfected with control siRNAs followed by stimulations with Rv2463 or infection with *M*. *tb* H37Rv, while bold lines represent cells transfected with specific siRNAs to indicated molecules followed by stimulations with Rv2463 or infection with *M*. *tb* H37Rv. Dotted line represents unstimulated or uninfected cells transfected with control siRNAs. One of three independent experiments is shown. P<0.015, control siRNA+Rv2463 v/s siMyD88+Rv2463; P<0.005, control siRNA+Rv2463 v/s siTRAF6+Rv2463; P<0.009, control siRNA+Rv2463 v/s siIRAK1+Rv2463. P values were calculated for 48h time point. Two-tailed Student’s t-test was employed for P values.

In order to investigate the receptors that would play a role in regulating CACNA1S expression, we investigated the role of the TLR pathway. To that end, we stimulated different TLRs with their known ligands (e.g. TLR2 was stimulated with Pam_3_Csk_4_; TLR4 was stimulated with LPS, TLR7 was stimulated with imiquimod and TLR9 was stimulated with CpG-DNA) in the presence or absence of stimulation with Rv2463 or infection with *M*. *tb* H37Rv. However, no significant effect of co-stimulation of any TLR on CACNA1S levels was observed either alone or in the presence of Rv2463 or *M*. *tb* stimulation (data not shown). This indicated that TLRs by themselves might not play a significant role towards CACNA1S expression during *M*. *tb* infection. Nevertheless, we investigated the role of the TLR pathway in regulating CACNA1S expression. To that end key intermediates in this pathway, namely, Myeloid Differentiation primary response 88 (MyD88), Interleukin-1 receptor-associated kinase 1 (IRAK1) and TNF receptor-associated factor 6 (TRAF6), were individually knockdown using specific siRNAs prior to stimulation with Rv2463 or infection with live *M*. *tb*. As shown in (Fig 4), knockdown of MyD88 had no significant effect on either Rv2463 or *M*. *tb* mediated CACNA1S expression. This indicated a minimal role for this molecule in CACNA1S expression. However, knockdown of either TRAF6 or IRAK1 significantly inhibited Rv2463 mediated but not *M*. *tb* mediated CACNA1S expression. This indicated that Rv2463 utilized TRAF6 and IRAK1 for CACNA1S expression. This also indicated that Rv2463 and *M*. *tb* stimulated different pathways for CACNA1S expression.

### Calcium regulates CACNA1S expression in macrophages

Internal and external calcium sources have different impact on various intracellular processes; internal or sequestered calcium from ER is released into the cytosol for creating a calcium burst. Cells require calcium from extracellular milieu during store depletion to fill the intracellular calcium reservoir. Therefore, in order to investigate if calcium homeostasis would also regulate CACNA1S expression during *M*. *tb* infection, using specific bio-pharmacological agents we inhibited internal calcium release and external calcium influx prior to stimulation with Rv2463 or infection with live *M*. *tb*. Internal calcium release was blocked using treatment with TMB8, a calcium antagonist that blocks intracellular calcium release [50], while external calcium influx was blocked using EGTA.

**Fig 5:**
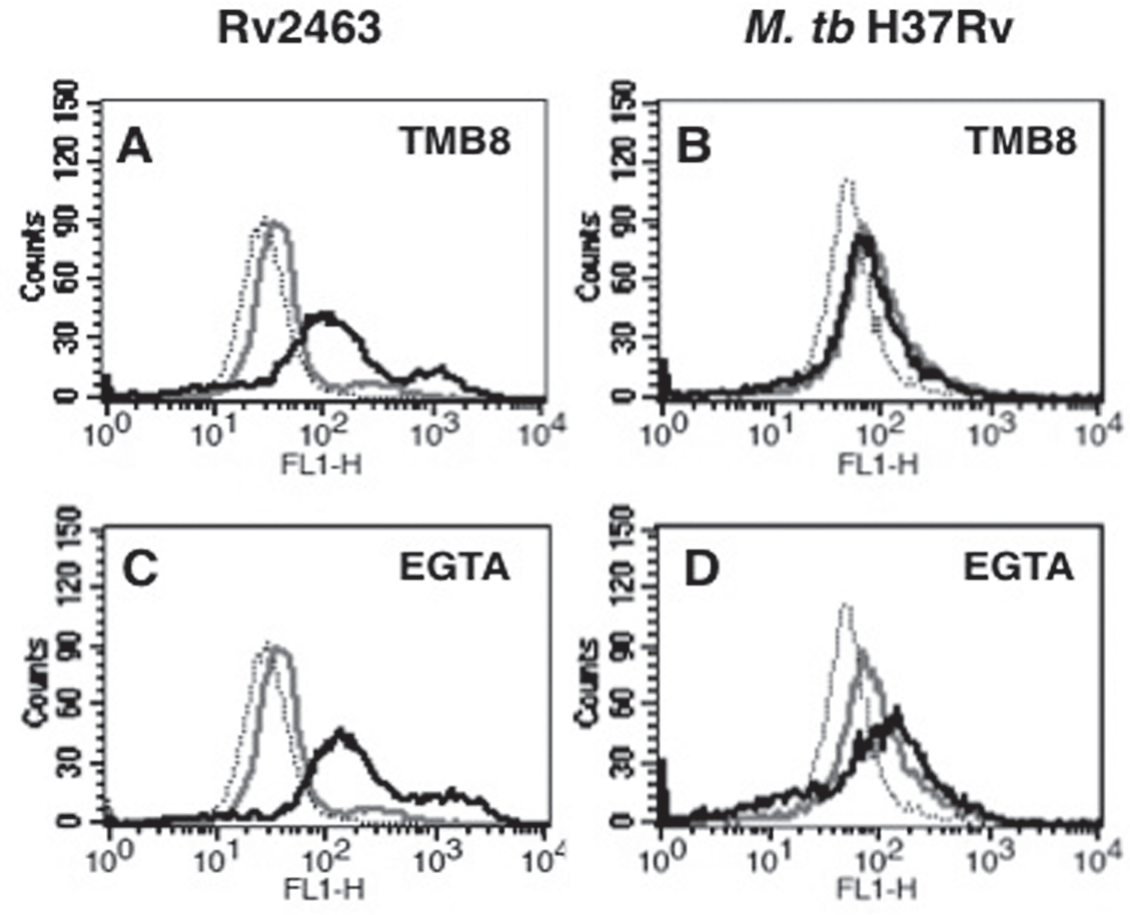
Calcium homeostasis regulates Rv2463 and *M*. *tb* mediated CACNA1S expression on macrophages. J774 cells were treated with inhibitors to internal calcium release (TMB8) or external calcium influx (EGTA) for 1h followed by stimulation with 25 μg/ml Rv2463 (panels A and C) or infection with 2 MOI of *M*. *tb* H37Rv (panels B and D) for 48h. CACNA1S expression was monitored by flow cytometry. Grey lines represent cells stimulated in the absence of inhibitors while bold lines represent cells stimulated in the presence of inhibitors. Dotted line represents unstimulated or uninfected cells. One of three independent experiments is shown. P<0.033 for macrophages+Rv2463 v/s macrophages+Rv2463+EGTA; P<0.019 for macrophages+Rv2463 v/s macrophages+Rv2463+TMB8. P<0.006 for macrophages+*M*. *tb* v/s macrophages+*M*. *tb*+EGTA; P<0.038 for macrophages + *M*. *tb* v/s macrophages+*M*. *tb*+TMB8; Two-tailed Student’s t-test was employed for P values.

As shown in (Fig 5A and 5C), inhibiting either internal calcium release or external calcium influx further increased CACNA1S expression on macrophages upon Rv2463 stimulation. This indicated that calcium homeostasis played a regulatory role during CACNA1S expression upon Rv2463 stimulation. On the contrary, macrophages infected with *M*. *tb* differentially regulated CACNA1S expression in the context of calcium homeostasis. Blocking calcium release from internal stores had no significant effect on CACNA1S expression in *M*. *tb* infected cells, while blocking external influx further upregulated CACNA1S expression as shown in (Fig 5B and 5D). The results also indicated that antigenic stimulation and *M*. *tb* infection differentially modulate CACNA1S expression with respect to calcium requirements. This could be attributed to the stimulation of different or multiple receptors in case of infection versus stimulation with fewer receptors during antigen stimulation.

### Internal calcium sensors STIM1 and STIM2 negatively regulate CACNA1S expression

**Fig 6:**
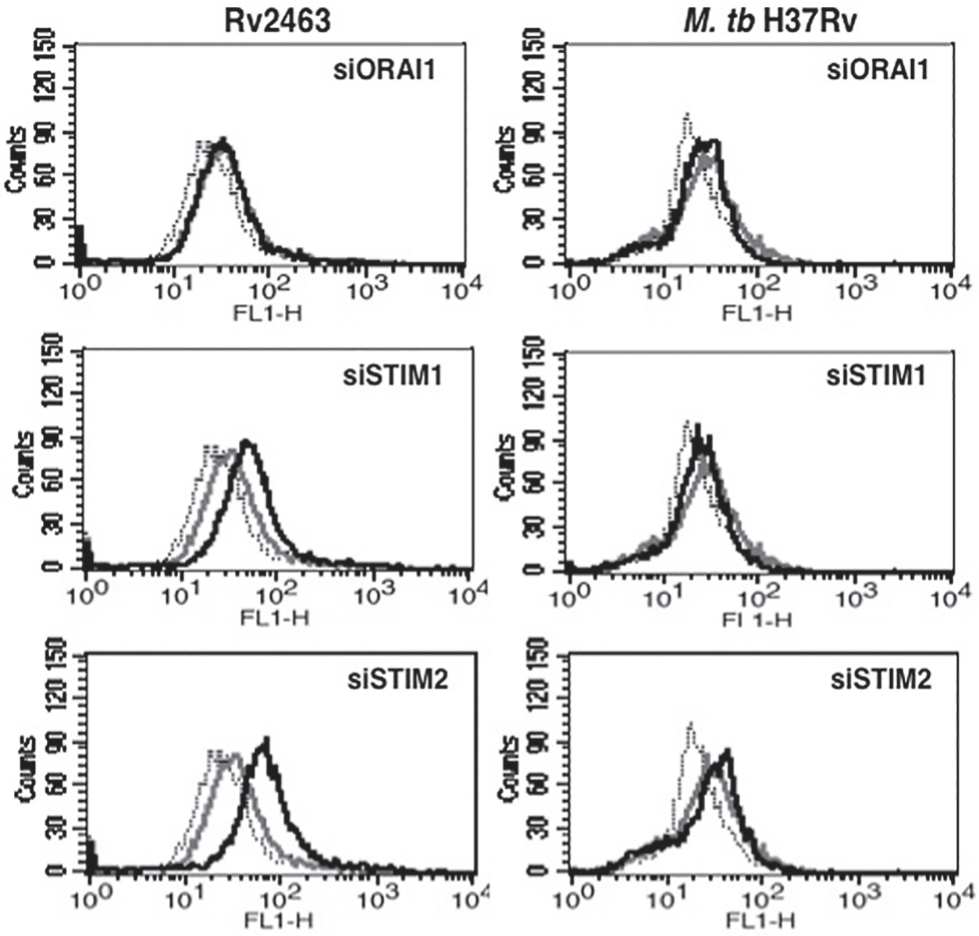
Molecular sensors of calcium homeostasis positively regulate Rv2463 mediated CACNA1S expression on macrophages. J774 cells were transfected with siRNAs against ORAI1, STIM1 or STIM2 for 36h followed by stimulations with 25 μg/ml Rv2463 or infection with 2 MOI of *M*. *tb* H37Rv for 48h. CACNA1S expression was monitored by flow cytometry. Grey lines represent cells transfected with control siRNAs followed by stimulations with Rv2463 or infection with *M*. *tb*. Bold lines represent cells transfected with specific siRNA to indicated molecules followed by stimulations with Rv2463 or infection with *M*. *tb*. Dotted lines represent unstimulated or uninfected cells transfected with control siRNAs. One of three independent experiments is shown. P<0.022 for control siRNA+Rv2463 v/s siSTIM1+ Rv2463; P<0.016 for control siRNA+Rv2463 v/s siSTIM2+Rv2463. Two-tailed Student’s t-test was employed for P values.

Store operated calcium channel /calcium release activated calcium channels are the major regulators of internal calcium reserves, such as the endoplasmic reticulum. The levels of calcium inside the endoplasmic reticulum are sensed by internal calcium sensors- Stromal Interaction Molecule 1 and 2 (STIM1 and STIM2) that together with Ca^2+^ release-activated Ca^2+^ channel (CRAC) subunit, ORAI1 regulate calcium homeostasis. Therefore, we investigated the role of these molecular sensors in regulating CACNA1S expression. As shown in (Fig 6), knockdown of STIM1 and STIM2 further upregulated Rv2463 mediated CACNA1S expression with no significant effects upon knockdown of ORAI1. Interestingly, knockdown of these sensors had no significant effect on *M*. *tb* induced CACNA1S expression despite a role of calcium influx in *M*. *tb* induced CACNA1S expression. This indicated that calcium homeostasis differentially regulated Rv2463 and *M*. *tb* induced CACNA1S expression. This could also indicate the involvement of different calcium regulators that govern CACNA1S expression during *M*. *tb* infection.

### Transcription factor CREB regulates CACNA1S expression during *M*. *tb* infection

**Fig 7:**
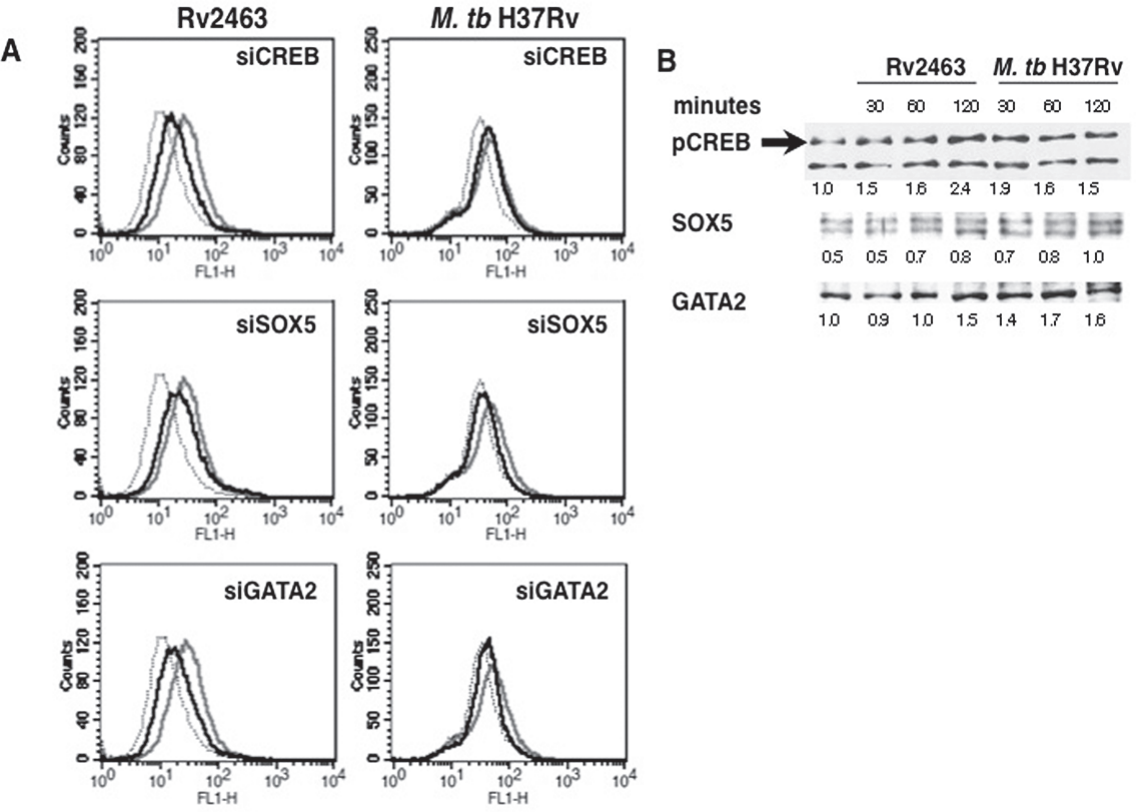
Transcription factors CREB, SOX5 and GATA2 regulate Rv2463 and *M*. *tb* mediated CACNA1S expression on macrophages. For Panel A, J774 cells were transfected with siRNAs against CREB or SOX5 or GATA2 for 36h followed by stimulations with 25 μg/ml Rv2463 or infection with 2 MOI of *M*. *tb* H37Rv for 48h. CACNA1S expression was monitored by flow cytometry. Grey lines represent cells transfected with control siRNAs followed by stimulations with Rv2463 or infection with *M*. *tb*. Bold lines represent cells transfected with specific siRNA to indicated molecules followed by stimulations with Rv2463 or infection with *M*. *tb* H37Rv. Dotted lines represent unstimulated cells transfected with control siRNAs. One of three independent experiments is shown. For Panel B, J774 cells were stimulated with 25 μg/ml Rv2463 or infection with 2 MOI of *M*. *tb* H37Rv for indicated times and nuclear extracts were western blotted for indicated molecules. Arrow indicates specific band. Numbers below the bands indicates relative intensities of the blots. One of two independent experiments is shown. For Panel A, P<0.035, control siRNA+Rv2463 v/s Creb+Rv2463; P<0.038 for control siRNA+Rv2463 v/s siSOX5+Rv2463; P<0.036 for control siRNA+Rv2463 v/s siGATA2+Rv2463. Two-tailed Student’s t-test was employed for P values. For Panel B, Creb phosphorylation; P<0.006, Unstimulated v/s Rv2463 stimulation 30 minutes; P<0.004, Unstimulated v/s Rv2463 stimulation 60 minutes; P<0.002, Unstimulated v/s Rv2463 stimulation 120 minutes; P<0.001, Unstimulated v/s *M*. *tb* infection 30 minutes P<0.003, Unstimulated v/s *M*. *tb* infection 60 minutes; P<0.004, Unstimulated v/s *M*. *tb* infection 120 minutes. Two-tailed Student’s t-test was employed for P values.

We also investigated the role of transcription factors CREB, SOX5 and GATA2 in regulating CACNA1S expression in J774 macrophages as the putative promoter elements of CACNA1S have binding sites for these molecules [49]. Calcium has been reported to regulate the activation of transcription factors under various conditions [51]. To that end, we knockdown CREB, SOX-5 and GATA-2 using specific siRNAs followed by Rv2463 stimulation or *M*. *tb* infection. As shown in (Fig 7A), knockdown of CREB, SOX5 or GATA2 significantly inhibited Rv2463 mediated CACNA1S expression. Knockdown of SOX5 and GATA2 but not CREB brought down the levels of *M*. *tb* induced CACNA1S. These data indicate the roles of these transcription factors in CACNA1S expression. We also investigated whether stimulation of macrophages with Rv2463 or infection with *M*. *tb* would modulate the activation of these transcription factors. As shown in (Fig 7B), stimulation with Rv2463 significantly activated phospho-CREB (pCREB) and to a lesser extent also activated GATA2 and SOX5. Infection with live *M*. *tb* also significantly activated pCREB but to a lesser extent when compared with Rv2463 mediated pCREB activation. *M*. *tb* infection also marginally activated GATA2 expression. Nevertheless, the collective results of (Fig 7) indicate putative roles for these transcription factors in mediating CACNA1S expression.

**Fig 8:**
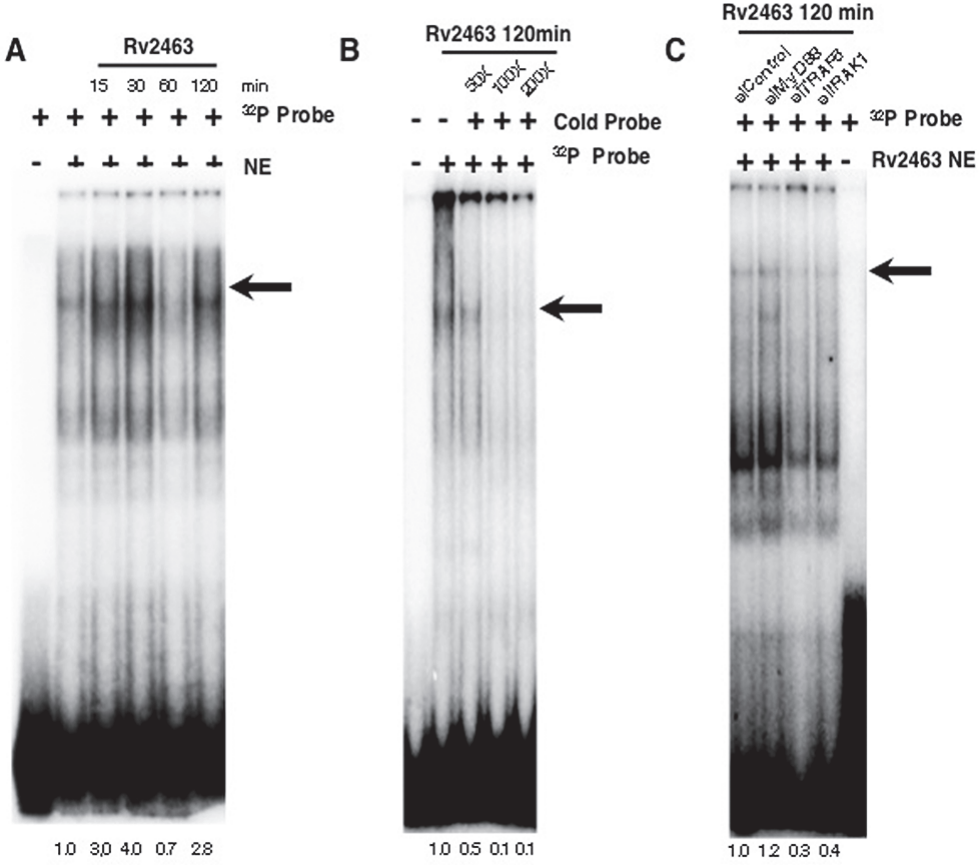
Rv2463 recruits CREB to CACNA1S promoter in a MyD88 independent and TLR dependent pathway. For Panel A, J774 cells were stimulated with 25 μg/ml Rv2463 for indicated times. Nuclear extracts (NE) were subjected to EMSA with ^32^P-labeled oligo (Probe) corresponding to 29 bp in the CACNA1S promoter. For Panel B, nuclear extracts from J774 cells stimulated with 25 μg/ml Rv2463 for 120 min were subjected to EMSA in the presence or absence of indicated concentrations of unlabelled oligo (Cold Probe). For Panel C, J774 cells were transfected with siRNAs to MyD88 or TRAF6 or IRAK1 for 36h followed by stimulations with 25 μg/ml Rv2463 for 120 min. siControl represents cells transfected with control siRNAs. Arrow indicates the specific band. One of three independent experiments is shown. P<0.003, Unstimulated v/s Rv2463 stimulation 15 minutes P<0.002, Unstimulated v/s Rv2463 stimulation 30 minutes; P<0.010, Unstimulated v/s Rv2463 stimulation 60 minutes; P<0.003, Unstimulated v/s Rv2463 stimulation 120 minutes.

We also investigated the dynamics of pCREB activation and DNA binding in the context of TLR pathway and calcium homeostasis. To that end we carried out electromobility shift assays to monitor the recruitment of pCREB to CACNA1S promoter sequence following stimulation of cells with Rv2463. As shown in (Fig 8A), stimulation with Rv2463 activated binding of pCREB to DNA in a time dependent manner. We ascertained the specificity of the shift by cold competition. As shown in (Fig 8B), cold competition with 100 fold molar excess of cold probe completely saturated the binding of ^32^P-labelled CREB probe indicating specificity of the band. Next we knockdown MyD88, TRAF6 or IRAK1 and carried out EMSA. As shown in (Fig 8C) and in agreement with the data in (Fig 4), knockdown of TRAF6 or IRAK1 but not MyD88 significantly inhibited pCREB binding to DNA thereby indicating that TRAF6 and IRAK1 activated pCREB for CACNA1S expression.

### Oxidative burst inhibits CACNA1S expression

**Fig 9:**
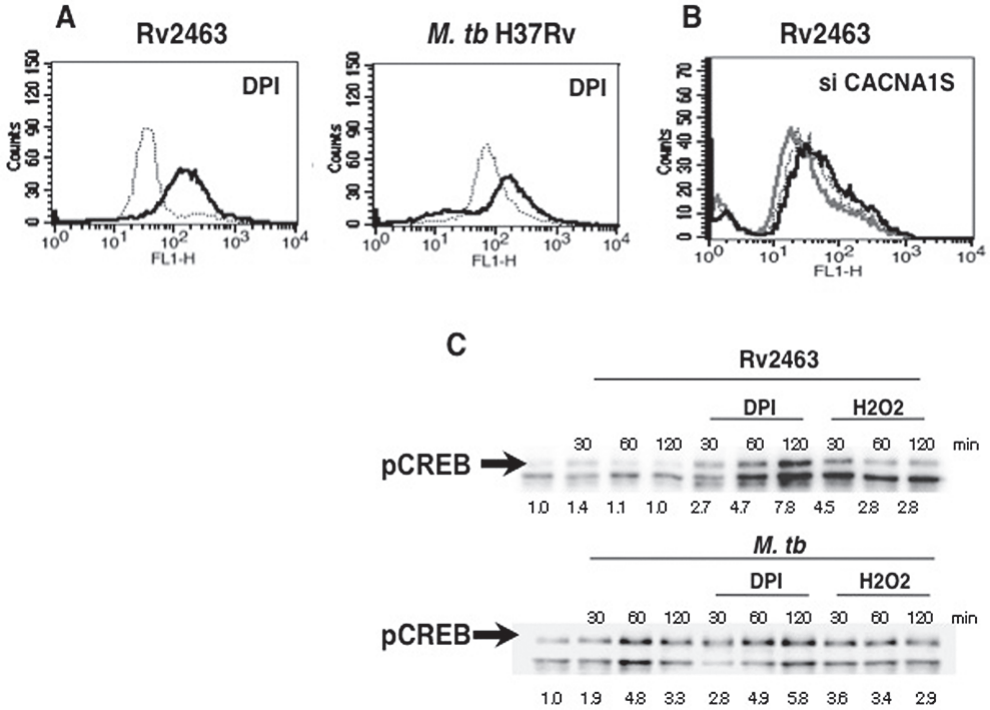
Cross regulation of CACNA1S, ROS and pCREB during Rv2463 stimulation or *M*. *tb* infection. For Panel A, J774 cells were stimulated with 25 μg/ml Rv2463 or infected with 2 MOI *M*. *tb* H37Rv for 48h in the presence (bold lines) or absence (dotted lines) of indicated inhibitors to ROS. CACNA1S levels were monitored by flow cytometry. For Panel B, J774 cells were transfected with siRNAs to CACNA1S for 36h followed by stimulation with 25 μg/ml Rv2463 for 48h. ROS levels were monitored by FACS. Grey line represents Rv2463 stimulated cells transfected with control siRNAs, while bold line represents Rv2463 stimulated cells transfected with siRNAs specific to CACNA1S. Dotted line represents unstimulated cells transfected with control siRNAs. For Panel C, J774 cells were stimulated with 25 μg/ml Rv2463 or infected with 2 MOI *M*. *tb* H37Rv for indicated times in the presence or absence of indicated inhibitors to ROS and nuclear extracts were probed for pCREB levels. Numbers below the blots indicate relative intensities of the bands. Arrow shows the specific band. Data from one of three experiments is shown. In Panel A, P<0.024 for Rv2463 v/s DPI+Rv2463; P<0.013 for *M*. *tb* v/s DPI+*M*. *tb*. In Panel B, P<0.006 for siControl+Rv2463 v/s siCACNA1S+Rv2463. In Panel C, at 120 minutes, P<0.024, for *M*. *tb* v/s DPI+*M*. *tb*. Two-tailed Student’s t-test was employed for P values.

We and others have previously reported the cross-regulation of Reactive Oxygen Species and calcium homeostasis [47]. Therefore, we next investigated if CACNA1S is also subject to this cross-regulation during *M*. *tb* infection. To that end, we scored for Rv2463 and *M*. *tb* mediated CACNA1S expression upon ROS inhibition. As shown in (Fig 9A), inhibiting ROS further enhanced CACNA1S expression upon Rv2463 stimulation and *M*. *tb* infection. This indicated that ROS played an inhibitory role during CACNA1S expression. These data are in agreement with our recent results wherein Rv2463 inhibited ROS generation in DCs as well as in macrophages [42, 46]. Next we investigated whether CACNA1S also regulated ROS generation. To that end, using specific siRNAs we knocked down CACNA1S and monitored ROS expression in the context of Rv2463 stimulation. As reported earlier stimulation with Rv2463 inhibited ROS levels below unstimulated controls, (Fig 9B), (compare dotted line with grey line). However, knockdown of CACNA1S reversed the effects of Rv2463 by increasing ROS levels (Fig 9B), (compare grey line with dark line). Owing to the unavailability of a flow cytometer in the BSL3 facility we could not carryout this experiment with *M*. *tb* H37Rv. Nevertheless, the results of (Fig 9A and 9B) clearly point towards an inhibitory role for ROS during CACNA1S expression and a functional significance of Rv2463 mediated inhibition of ROS generation.

Next, we also investigated the regulation of ROS with pCREB activation. To that end we monitored pCREB activation upon ROS inhibition in the context of Rv2463 stimulation. As shown in (Fig 9C), inhibiting ROS further activated Rv2463 mediated pCREB in macrophages and to a lesser extent in *M*. *tb* infected macrophages. This once again pointed towards an inhibitory role for ROS in regulation of pCREB activation. Put together, the results in (Fig 9) point towards an interesting interplay between ROS, pCREB and CACNA1S expression during Rv2463 stimulation.

## Discussion

Calcium plays an essential role in mediating physiological pathways in a cell [52]. Oscillations in calcium levels regulate the activation of many second messengers and transcription factors in response to receptor triggering [53]. Consequently, regulation of calcium dynamics, particularly during acute and chronic infections has determinant effects on the outcome of immune responses to a pathogenic challenge. With respect to *M*. *tb* infection, calcium has been shown to impart immune activation and immune suppression in macrophages and DCs [54, 55]. Maturation and fusion of phagosomes with lysosomes following *M*. *tb* infection is highly dependent on the calcium calcineurin pathway. Calcinuerin regulates the expression of coronin-1 on phagosomes thus affecting phagosome maturation [56]. VGCC plays different roles during cellular physiological and neurological conditions; its role in mediating immune activation/suppression has recently surfaced [57].

We previously demonstrated an inhibitory role for VGCC during *M*. *tb* pathogenesis [37]. Blocking L-type or R-type VGCC induced calcium influx in infected cells, activated pro-inflammatory and protective responses from DCs and PBMCs and restricted *M*. *tb* growth within cells and in infected mice. Patients with active tuberculosis expressed high levels of VGCC on their PBMCs that were reduced following chemotherapy.

In parallel, we enriched antigens that were expressed in infected macrophages as a function of time [42]. Of these antigens, Rv2463 was expressed within 24h of infection and induced suppressor responses. Therefore, for this study we envisaged that Rv2463 could regulate the expression levels of CACNA1S. Our results point to unique and novel mechanisms of CACNA1S expression by Rv2463 and live *M*. *tb* infection. Our previous results and that of others have shown that many responses mediated by *M*. *tb* can be reproduced in principle by their antigens. We have extensively characterized the role of Culture Filtrate Protein-10 (CFP-10) and Early Secreted Antigenic Target (ESAT6) in regulating immune responses from DCs. Both these antigens induce the differentiation of DCs from bone marrow precursors [58]. These antigen differentiated DCs induce suppressor responses to *M*. *tb* [59, 60]. Therefore, the present data are in concurrence with our earlier results with respect to antigen mediated modulation of immune responses.

Although TLR pathway in general has been shown to induce protective responses to many pathogens including *M*. *tb* [61–63] however, many reports also indicate that microbes and/or their antigens use the TLR pathway to reverse protective into suppressive responses. For example, the *M*. *tb* 19 kDa lipoprotein utilizes the TLR2 pathway to subvert Interferon-γ induced responses [64]. Our data add support to the above studies and provide another example of *M*. *tb* utilizing the TLR pathway in facilitating suppressor responses.

While calcium from internal and external source played an inhibitory role for Rv2463 mediated CACNA1S expression, only calcium from external stores had an effect with respect to CACNA1S expression upon *M*. *tb* infection with no role for the calcium from internal stores. Calcium is an important second messenger that regulates the activity of transcription factors. The fact that CREB, GATA2 and SOX5 also played differential regulatory roles in Rv2463 or *M*. *tb* induced CACNA1S expression, this points to a link between the routing of calcium from external v/s internal sources during Rv2463 or *M*. *tb* infection and the activation of the transcription factors that differentially govern CACNA1S expression. We also established a cross-regulation of ROS and CACNA1S expression with respect to Rv2463 stimulation. ROS and calcium are known to cross-regulate each other [65] and our data adds further support to this relationship.

**Fig 10:**
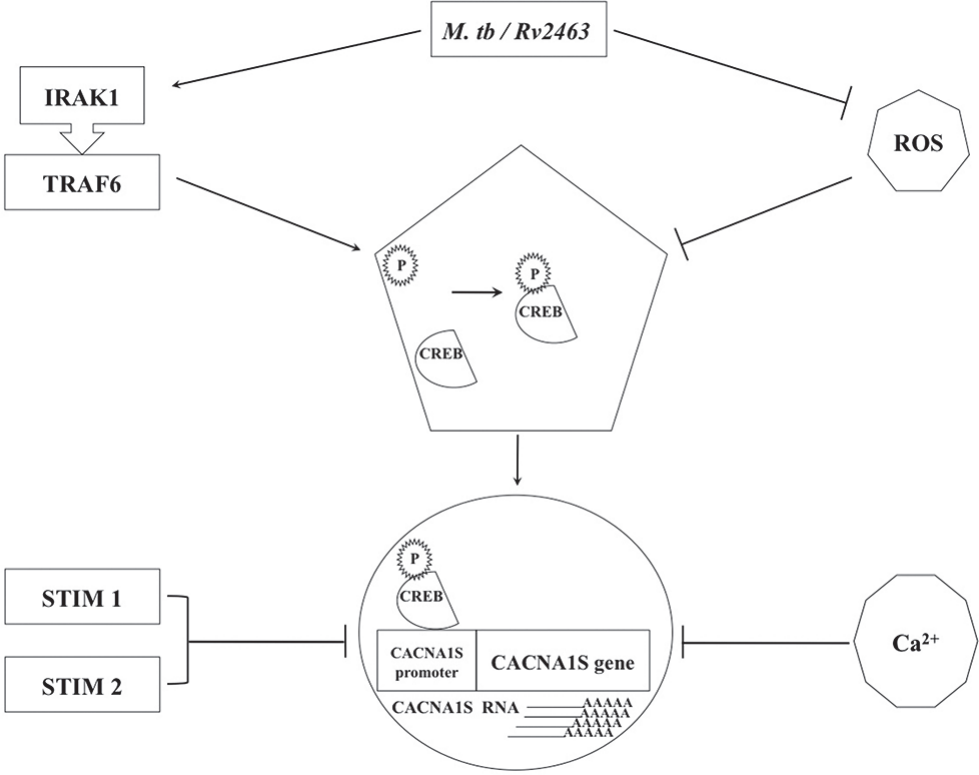
Proposed model for CACNA1S expression by *M*. *tb*. Stimulation of macrophages by *M*. *tb* or Rv2463 increases CREB phosphorylation in a TLR dependent MyD88 independent pathway that involves IRAK1 and TRAF6. Phosphorylated CREB (p-CREB) translocates to the nucleus and binds to CACNA1S promoter to induce CACNA1S protein expression. In parallel mycobacterial stimulation attenuates the generation of reactive oxygen species (ROS) that further contributes to CREB phosphorylation. Calcium homeostasis sensors STIM1 and STIM2 also play an inhibitory role in CACNA1S upregulation upon *M*. *tb* infection or Rv2463 stimulation.

Put together, and as depicted in (Fig 10), our data indicate that Rv2463 and live *M*. *tb* infection induce higher expression of CACNA1S in macrophages by upregulating CREB phosphorylation in a TLR dependent MyD88 independent pathway that involves IRAK1 and TRAF6. In parallel attenuation of reactive oxygen species (ROS) generation upon mycobacterial stimulation further contributes to CREB phosphorylation. Differential calcium homeostasis as mediated by the action of sensors STIM1 and STIM2 also also contributes to CACNA1S upregulation upon *M*. *tb* infection or Rv2463 stimulation. This pathway could result from engagement of either single (e.g. in case of Rv2463 stimulation) or multiple receptors (e.g. in the case of *M*. *tb* infection) thereby differentially activating components of the pathway that collectively regulate CACNA1S expression.

## Supporting Information

S1 Fig Transfection efficiency of molecules. J774 cells were transfected with either control siRNAs (siControl) or siRNAs to indicated molecules and cytoplasmic or nuclear extracts were prepared as described in Materials and Methods. The extracts were resolved on SDS-PAGE and western blotted for indicated molecules.
(DOC)

S2 Fig J774 macrophages express CACNA1S and Rv2463 induced CACNA1S expression is specific. For Panel A, CACNA1S expression was checked on J774 cells by flow cytometry. Dotted lines represent unstained cells, thin line represents cells stained with isotype matched control antibody and bold line represents cells stained with antibody specific to CACNA1S. For Panel B, J774 cells were stimulated with 25 μg/ml Rv2463 for 48h and CACNA1S expression was monitored by flow cytometry. Dotted line represents unstimulated cells, thin line represents Rv2463 stimulated cells stained with isotype matched control antibody, while the bold line represents Rv2463 stimulated cells stained with CACNA1S specific antibody.
(DOC)

S3 Fig Rv3416 does not induce CACNA1S on macrophages. J774 cells were stimulated with 25 μg/ml Rv3416 for 48h and CACNA1S expression was monitored by flow cytometry. Shaded histogram represents unstained cells, dotted line represents unstimulated cells stained with CACNA1S specific antibody while the bold line represents Rv3416 stimulated cells stained with CACNA1S specific antibody.
(DOC)

S4 Fig Rv2463 is internalized by J774 macrophages. J774 cells were incubated with PE-streptavidin-biotin conjugated Rv2463 (25 μg/ml). Internalization of the antigen was monitored using confocal microscopy. Images show Z-stacks of 1μm section for Rv3416 at 30 min post-stimulation.
(DOC)

S5 Fig Rv2463 and *M*. *tb* induce the upregulation of CACNA1S on bone marrow derived macrophages. Mouse bone marrow derived macrophages were stimulated with Rv2463 at 25 μg/ml or infecected with 2 MOI *M*. *tb* H37Rv for 48h. CACNA1S expression on cell surface was monitored by flow cytometry. Bold lines represent stimulations with Rv2463 (left panel) or *M*. *tb* H37Rv (right panel) and thin lines represent unstimulated controls. Data from one of two independent experiments are shown.
(DOC)

## References

[1] Jassal MS, Bishai WR (2010) Epidemiology and challenges to the elimination of global tuberculosis. Clin Infect Dis 50 Suppl 3: S156–164. 10.1086/651486 20397943 PMC4575217

[2] WHO Global tuberculosis report. Available: http://apps.who.int/iris/bitstream/10665/91355/1/9789241564656_eng.pdf?ua=1. Accessed 16 October 2014.

[3] Flynn JL (2004) Immunology of tuberculosis and implications in vaccine development. Tuberculosis (Edinb) 84: 93–101. 14670350 10.1016/j.tube.2003.08.010

[4] Foote S (1999) Mediating immunity to mycobacteria. Nat Genet 21: 345–346. 10192376 10.1038/7663

[5] Flynn JL, Chan J (2001) Immunology of tuberculosis. Annu Rev Immunol 19: 93–129. 11244032 10.1146/annurev.immunol.19.1.93

[6] Malik ZA, Thompson CR, Hashimi S, Porter B, Iyer SS, Kusner DJ (2003) Cutting edge: Mycobacterium tuberculosis blocks Ca2+ signaling and phagosome maturation in human macrophages via specific inhibition of sphingosine kinase. J Immunol 170: 2811–2815. 12626530 10.4049/jimmunol.170.6.2811

[7] Balcewicz-Sablinska MK, Keane J, Kornfeld H, Remold HG (1998) Pathogenic Mycobacterium tuberculosis evades apoptosis of host macrophages by release of TNF-R2, resulting in inactivation of TNF-alpha. J Immunol 161: 2636–2641. 9725266

[8] Shin DM, Jeon BY, Lee HM, Jin HS, Yuk JM, Song CH, et al (2010) Mycobacterium tuberculosis eis regulates autophagy, inflammation, and cell death through redox-dependent signaling. PLoS Pathog 6: e1001230 10.1371/journal.ppat.1001230 21187903 PMC3002989

[9] Singhal A, Jaiswal A, Arora VK and Prasad HK (2007) Modulation of gamma interferon receptor 1 by Mycobacterium tuberculosis: a potential immune response evasive mechanism. Infect Immun 75: 2500–2510. 17339358 10.1128/IAI.01743-06 PMC1865798

[10] Morris SA, Tanowitz H, Hatcher V, Bilezikian JP, Wittner M (1988) Alterations in intracellular calcium following infection of human endothelial cells with Trypanosoma cruzi. Mol Biochem Parasitol 29: 213–221. 3045542 10.1016/0166-6851(88)90076-x

[11] Wadsworth SJ, Goldfine H (1999) Listeria monocytogenes phospholipase C-dependent calcium signaling modulates bacterial entry into J774 macrophage-like cells. Infect Immun 67: 1770–1778. 10085017 10.1128/iai.67.4.1770-1778.1999 PMC96527

[12] Koul A, Herget T, Klebl B, Ullrich A (2004) Interplay between mycobacteria and host signalling pathways. Nat Rev Microbiol 2: 189–202. 15083155 10.1038/nrmicro840

[13] Jaconi ME, Lew DP, Carpentier JL, Magnusson KE, Sjogren M, Stendahl O (1990) Cytosolic free calcium elevation mediates the phagosome-lysosome fusion during phagocytosis in human neutrophils. J Cell Biol 110: 1555–1564. 2110568 10.1083/jcb.110.5.1555 PMC2200167

[14] Yano S, Tokumitsu H, Soderling TR (1998) Calcium promotes cell survival through CaM-K kinase activation of the protein-kinase-B pathway. Nature 396: 584–587. 9859994 10.1038/25147

[15] Dolmetsch RE, Lewis RS, Goodnow CC, Healy JI (1997) Differential activation of transcription factors induced by Ca2+ response amplitude and duration. Nature 386: 855–858. 9126747 10.1038/386855a0

[16] Cantrell D (1996) T cell antigen receptor signal transduction pathways. Annu Rev Immunol 14: 259–274. 8717515 10.1146/annurev.immunol.14.1.259

[17] Deisseroth K, Bito H, Tsien RW (1996) Signaling from synapse to nucleus: postsynaptic CREB phosphorylation during multiple forms of hippocampal synaptic plasticity. Neuron 16: 89–101. 8562094 10.1016/s0896-6273(00)80026-4

[18] Hsu LC, Park JM, Zhang K, Luo JL, Maeda S, Kaufman RJ, et al (2004) The protein kinase PKR is required for macrophage apoptosis after activation of Toll-like receptor 4. Nature 428: 341–345. 15029200 10.1038/nature02405

[19] Park JM, Greten FR, Wong A, Westrick RJ, Arthur JS, Otsu K, et al (2005) Signaling pathways and genes that inhibit pathogen-induced macrophage apoptosis—CREB and NF-kappaB as key regulators. Immunity 23: 319–329. 16169504 10.1016/j.immuni.2005.08.010

[20] Agarwal N, Lamichhane G, Gupta R, Nolan S, Bishai WR (2009) Cyclic AMP intoxication of macrophages by a Mycobacterium tuberculosis adenylate cyclase. Nature 460: 98–102. 10.1038/nature08123 19516256

[21] Berridge MJ (1993) Cell signalling. A tale of two messengers. Nature 365: 388–389. 8413581 10.1038/365388a0

[22] Lewis RS (2007) The molecular choreography of a store-operated calcium channel. Nature 446: 284–287. 17361175 10.1038/nature05637

[23] Catterall WA (2000) Structure and regulation of voltage-gated Ca2+ channels. Annu Rev Cell Dev Biol 16: 521–555. 11031246 10.1146/annurev.cellbio.16.1.521

[24] Nejatbakhsh N, Feng ZP (2011) Calcium binding protein-mediated regulation of voltage-gated calcium channels linked to human diseases. Acta Pharmacol Sin 32: 741–748. 10.1038/aps.2011.64 21642945 PMC4009971

[25] Catterall WA (2011) Voltage-gated calcium channels. Cold Spring Harb Perspect Biol 3: a003947 10.1101/cshperspect.a003947 21746798 PMC3140680

[26] Mu TW, Fowler DM, Kelly JW (2008) Partial restoration of mutant enzyme homeostasis in three distinct lysosomal storage disease cell lines by altering calcium homeostasis. PLoS Biol 6: e26 10.1371/journal.pbio.0060026 18254660 PMC2225441

[27] Wieland H, Hechtel N, Faigle M, Neumeister B (2006) Efficient intracellular multiplication of Legionella pneumophila in human monocytes requires functional host cell L-type calcium channels. FEMS Immunol Med Microbiol 47: 296–301. 16831218 10.1111/j.1574-695X.2006.00092.x

[28] Matza D, Badou A, Kobayashi KS, Goldsmith-Pestana K, Masuda Y, Komuro A, et al (2008) A scaffold protein, AHNAK1, is required for calcium signaling during T cell activation. Immunity 28: 64–74. 10.1016/j.immuni.2007.11.020 18191595 PMC2350190

[29] Poggi A, Rubartelli A, Zocchi MR (1998) Involvement of dihydropyridine-sensitive calcium channels in human dendritic cell function. Competition by HIV-1 Tat. J Biol Chem 273: 7205–7209. 9516412 10.1074/jbc.273.13.7205

[30] Rosales C, Brown EJ (1992) Calcium channel blockers nifedipine and diltiazem inhibit Ca2+ release from intracellular stores in neutrophils. J Biol Chem 267: 1443–1448. 1730694

[31] Zocchi MR, Rubartelli A, Morgavi P, Poggi A (1998) HIV-1 Tat inhibits human natural killer cell function by blocking L-type calcium channels. J Immunol 161: 2938–2943. 9743356

[32] Kotturi MF, Carlow DA, Lee JC, Ziltener HJ, Jefferies WA (2003) Identification and functional characterization of voltage-dependent calcium channels in T lymphocytes. J Biol Chem 278: 46949–46960. 12954628 10.1074/jbc.M309268200

[33] Stokes L, Gordon J, Grafton G (2004) Non-voltage-gated L-type Ca2+ channels in human T cells: pharmacology and molecular characterization of the major alpha pore-forming and auxiliary beta-subunits. J Biol Chem 279: 19566–19573. 14981074 10.1074/jbc.M401481200

[34] Savignac M, Badou A, Moreau M, Leclerc C, Guery JC, Paulet P, et al (2001) Protein kinase C-mediated calcium entry dependent upon dihydropyridine sensitive channels: a T cell receptor-coupled signaling pathway involved in IL-4 synthesis. FASEB J 15: 1577–1579. 11427491 10.1096/fj.00-0733fje

[35] Badou A, Savignac M, Moreau M, Leclerc C, Pasquier R, Druet P, et al (1997) HgCl2-induced interleukin-4 gene expression in T cells involves a protein kinase C-dependent calcium influx through L-type calcium channels. J Biol Chem 272: 32411–32418. 9405450 10.1074/jbc.272.51.32411

[36] Grafton G, Stokes L, Toellner KM, Gordon J (2003) A non-voltage-gated calcium channel with L-type characteristics activated by B cell receptor ligation. Biochem Pharmacol 66: 2001–2009. 14599558 10.1016/j.bcp.2003.07.005

[37] Gupta S, Salam N, Srivastava V, Singla R, Behera D, Khayyam KU, et al (2009) Voltage gated calcium channels negatively regulate protective immunity to Mycobacterium tuberculosis. PLoS One 4: e5305 10.1371/journal.pone.0005305 19390594 PMC2669286

[38] Gupta S, Tyagi S, Almeida DV, Maiga MC, Ammerman NC, Bishai WR Acceleration of tuberculosis treatment by adjunctive therapy with verapamil as an efflux inhibitor. Am J Respir Crit Care Med 188: 600–607. 10.1164/rccm.201304-0650OC 23805786 PMC3827702

[39] Adams KN, Takaki K, Connolly LE, Wiedenhoft H, Winglee K, Humbert O, et al Drug tolerance in replicating mycobacteria mediated by a macrophage-induced efflux mechanism. Cell 145: 39–53. 10.1016/j.cell.2011.02.022 21376383 PMC3117281

[40] Nagabhushanam V, Solache A, Ting LM, Escaron CJ, Zhang JY, Ernst JD (2003) Innate inhibition of adaptive immunity: Mycobacterium tuberculosis-induced IL-6 inhibits macrophage responses to IFN-gamma. J Immunol 171: 4750–4757. 14568951 10.4049/jimmunol.171.9.4750

[41] Podinovskaia M, Lee W, Caldwell S, Russell DG (2013) Infection of macrophages with Mycobacterium tuberculosis induces global modifications to phagosomal function. Cell Microbiol 15: 843–859. 10.1111/cmi.12092 23253353 PMC3620910

[42] Gupta D, Sharma S, Singhal J, Satsangi AT, Antony C, Natarajan K (2010) Suppression of TLR2-induced IL-12, reactive oxygen species, and inducible nitric oxide synthase expression by Mycobacterium tuberculosis antigens expressed inside macrophages during the course of infection. J Immunol 184: 5444–5455. 10.4049/jimmunol.0903283 20385877

[43] Badou A, Jha MK, Matza D, Mehal WZ, Freichel M, Flockerzi V, et al (2006) Critical role for the beta regulatory subunits of Cav channels in T lymphocyte function. Proc Natl Acad Sci U S A 103: 15529–15534. 17028169 10.1073/pnas.0607262103 PMC1622857

[44] Matza D, Flavell RA (2009) Roles of Ca(v) channels and AHNAK1 in T cells: the beauty and the beast. Immunol Rev 231: 257–264. 10.1111/j.1600-065X.2009.00805.x 19754902

[45] Jha MK, Badou A, Meissner M, McRory JE, Freichel M, Flockerzi V, et al (2009) Defective survival of naive CD8+ T lymphocytes in the absence of the beta3 regulatory subunit of voltage-gated calcium channels. Nat Immunol 10: 1275–1282. 10.1038/ni.1793 19838200 PMC2785134

[46] Singhal J, Agrawal N, Vashishta M, Priya NG, Tiwari BK, Singh Y, et al (2012) Suppression of dendritic cell-mediated responses by genes in calcium and cysteine protease pathways during Mycobacterium tuberculosis infection. J Biol Chem 287: 11108–11121 10.1074/jbc.M111.300319 22337888 PMC3322814

[47] Selvakumar A, Antony C, Singhal J, Tiwari BK, Singh Y, Natarajan K (2014) Reciprocal regulation of reactive oxygen species and phospho-CREB regulates voltage gated calcium channel expression during Mycobacterium tuberculosis infection. PLoS One 9: e96427 10.1371/journal.pone.0096427 24797940 PMC4010530

[48] Salam N, Gupta S, Sharma S, Pahujani S, Sinha A, Saxena RK, et al (2008) Protective immunity to Mycobacterium tuberculosis infection by chemokine and cytokine conditioned CFP-10 differentiated dendritic cells. PLoS One 3: e2869 10.1371/journal.pone.0002869 18682728 PMC2478708

[49] Zheng Z, Wang ZM, Delbono O (2002) Charge movement and transcription regulation of L-type calcium channel alpha(1S) in skeletal muscle cells. J Physiol 540: 397–409. 11956331 10.1113/jphysiol.2001.013464 PMC2290248

[50] Dougherty RW, Niedel JE (1986) Cytosolic calcium regulates phorbol diester binding affinity in intact phagocytes. J Biol Chem 261: 4097–4100. 3081515

[51] Mellstrom B, Savignac M, Gomez-Villafuertes R, Naranjo JR (2008) Ca2+-operated transcriptional networks: molecular mechanisms and in vivo models. Physiol Rev 88: 421–449. 10.1152/physrev.00041.2005 18391169

[52] Petersen OH, Petersen CC, Kasai H (1994) Calcium and hormone action. Annu Rev Physiol 56: 297–319. 8010742 10.1146/annurev.ph.56.030194.001501

[53] Feske S (2007) Calcium signalling in lymphocyte activation and disease. Nat Rev Immunol 7: 690–702. 17703229 10.1038/nri2152

[54] Liu W, Matsumori A (2011) Calcium channel blockers and modulation of innate immunity. Curr Opin Infect Dis 24: 254–258. 10.1097/QCO.0b013e3283463e5b 21467929

[55] Faries MB, Bedrosian I, Xu S, Koski G, Roros JG, Moise MA, et al (2001) Calcium signaling inhibits interleukin-12 production and activates CD83(+) dendritic cells that induce Th2 cell development. Blood 98: 2489–2497. 11588047 10.1182/blood.v98.8.2489

[56] Jayachandran R, Sundaramurthy V, Combaluzier B, Mueller P, Korf H, Huygen K, et al (2007) Survival of mycobacteria in macrophages is mediated by coronin 1-dependent activation of calcineurin. Cell 130: 37–50. 17632055 10.1016/j.cell.2007.04.043

[57] Azenabor AA, Chaudhry AU (2003) Effective macrophage redox defense against Chlamydia pneumoniae depends on L-type Ca2+ channel activation. Med Microbiol Immunol 192: 99–106. 12736823 10.1007/s00430-002-0164-8

[58] Latchumanan VK, Singh B, Sharma P, Natarajan K (2002) Mycobacterium tuberculosis antigens induce the differentiation of dendritic cells from bone marrow. J Immunol 169: 6856–6864. 12471118 10.4049/jimmunol.169.12.6856

[59] Natarajan K, Latchumanan VK, Singh B, Singh S, Sharma P (2003) Down-regulation of T helper 1 responses to mycobacterial antigens due to maturation of dendritic cells by 10-kDa mycobacterium tuberculosis secretory antigen. J Infect Dis 187: 914–928. 12660938 10.1086/368173

[60] Balkhi MY, Latchumanan VK, Singh B, Sharma P, Natarajan K (2004) Cross-regulation of CD86 by CD80 differentially regulates T helper responses from Mycobacterium tuberculosis secretory antigen-activated dendritic cell subsets. J Leukoc Biol 75: 874–883. 14966193 10.1189/jlb.1003476

[61] Torres D, Barrier M, Bihl F, Quesniaux VJ, Maillet I, Akira S, et al (2004) Toll-like receptor 2 is required for optimal control of Listeria monocytogenes infection. Infect Immun 72: 2131–2139. 15039335 10.1128/IAI.72.4.2131-2139.2004 PMC375211

[62] Carmona J, Cruz A, Moreira-Teixeira L, Sousa C, Sousa J, Osorio NS, et al (2013) Strains Are Differentially Recognized by TLRs with an Impact on the Immune Response. PLoS One 8: e67277 23840651 10.1371/journal.pone.0067277 PMC3693941

[63] Harris TH, Mansfield JM, Paulnock DM (2007) CpG oligodeoxynucleotide treatment enhances innate resistance and acquired immunity to African trypanosomes. Infect Immun 75: 2366–2373. 17339353 10.1128/IAI.01649-06 PMC1865757

[64] Pennini ME, Pai RK, Schultz DC, Boom WH, Harding CV (2006) Mycobacterium tuberculosis 19-kDa lipoprotein inhibits IFN-gamma-induced chromatin remodeling of MHC2TA by TLR2 and MAPK signaling. J Immunol 176: 4323–4330. 16547269 10.4049/jimmunol.176.7.4323

[65] Sinha A, Singh A, Satchidanandam V, Natarajan K (2006) Impaired generation of reactive oxygen species during differentiation of dendritic cells (DCs) by Mycobacterium tuberculosis secretory antigen (MTSA) and subsequent activation of MTSA-DCs by mycobacteria results in increased intracellular survival. J Immunol 177: 468–478. 16785544 10.4049/jimmunol.177.1.468

