# Supplementary material for: Regulation of L-type Voltage Gated Calcium Channel CACNA1S in Macrophages upon Mycobacterium tuberculosis Infection
Source: PLoS One. 2015 Apr 27;10(4):e0124263. doi: 10.1371/journal.pone.0124263 (PMC4411123; doi:10.1371/journal.pone.0124263)
Supplement: S1 Fig — J774 cells were transfected with either control siRNAs (siControl) or siRNAs to indicated molecules and cytoplasmic or nuclear extracts were prepared as described in Materials and Methods. The extracts were resolved on SDS-PAGE and western blotted for indicated molecules. (DOC) [file pone.0124263.s001.doc]

**S1 Fig. Transfection efficiency of molecules.** J774 cells were transfected with either control siRNAs (siControl) or siRNAs to indicated molecules and cytoplasmic or nuclear extracts were prepared as described in Materials and Methods. The extracts were resolved on SDS-PAGE and western blotted for indicated molecules.
