# Supplementary material for: Regulation of L-type Voltage Gated Calcium Channel CACNA1S in Macrophages upon Mycobacterium tuberculosis Infection
Source: PLoS One. 2015 Apr 27;10(4):e0124263. doi: 10.1371/journal.pone.0124263 (PMC4411123; doi:10.1371/journal.pone.0124263)
Supplement: S2 Fig — For Panel A, CACNA1S expression was checked on J774 cells by flow cytometry. Dotted lines represent unstained cells, thin line represents cells stained with isotype matched control antibody and bold line represents cells stained with antibody specific to CACNA1S. For Panel B, J774 cells were stimulated with 25 μg/ml Rv2463 for 48h and CACNA1S expression was monitored by flow cytometry. Dotted line represents unstimulated cells, thin line represents Rv2463 stimulated cells stained with isotype matched control antibody, while the bold line represents Rv2463 stimulated cells stained with CACNA1S specific antibody. (DOC) [file pone.0124263.s002.doc]

**S2 Fig. J774 macrophages express CACNA1S and Rv2463 induced CACNA1S expression is specific**. For Panel A, CACNA1S expression was checked on J774 cells by flow cytometry. Dotted lines represent unstained cells, thin line represents cells stained with isotype matched control antibody and bold line represents cells stained with antibody specific to CACNA1S. For Panel B, J774 cells were stimulated with 25μg/ml Rv2463 for 48h and CACNA1S expression was monitored by flow cytometry. Dotted line represents unstimulated cells, thin line represents Rv2463 stimulated cells stained with isotype matched control antibody, while the bold line represents Rv2463 stimulated cells stained with CACNA1S specific antibody.
