# Supplementary material for: Regulation of L-type Voltage Gated Calcium Channel CACNA1S in Macrophages upon Mycobacterium tuberculosis Infection
Source: PLoS One. 2015 Apr 27;10(4):e0124263. doi: 10.1371/journal.pone.0124263 (PMC4411123; doi:10.1371/journal.pone.0124263)
Supplement: S3 Fig — J774 cells were stimulated with 25 μg/ml Rv3416 for 48h and CACNA1S expression was monitored by flow cytometry. Shaded histogram represents unstained cells, dotted line represents unstimulated cells stained with CACNA1S specific antibody while the bold line represents Rv3416 stimulated cells stained with CACNA1S specific antibody. (DOC) [file pone.0124263.s003.doc]

**S3 Fig. Rv3416 does not induce CACNA1S on macrophages**. J774 cells were stimulated with 25μg/ml Rv3416 for 48h and CACNA1S expression was monitored by flow cytometry. Shaded histogram represents unstained cells, dotted line represents unstimulated cells stained with CACNA1S specific antibody while the bold line represents Rv3416 stimulated cells stained with CACNA1S specific antibody.
