# Supplementary material for: Regulation of L-type Voltage Gated Calcium Channel CACNA1S in Macrophages upon Mycobacterium tuberculosis Infection
Source: PLoS One. 2015 Apr 27;10(4):e0124263. doi: 10.1371/journal.pone.0124263 (PMC4411123; doi:10.1371/journal.pone.0124263)
Supplement: S4 Fig — J774 cells were incubated with PE-streptavidin-biotin conjugated Rv2463 (25 μg/ml). Internalization of the antigen was monitored using confocal microscopy. Images show Z-stacks of 1μm section for Rv3416 at 30 min post-stimulation. (DOC) [file pone.0124263.s004.doc]

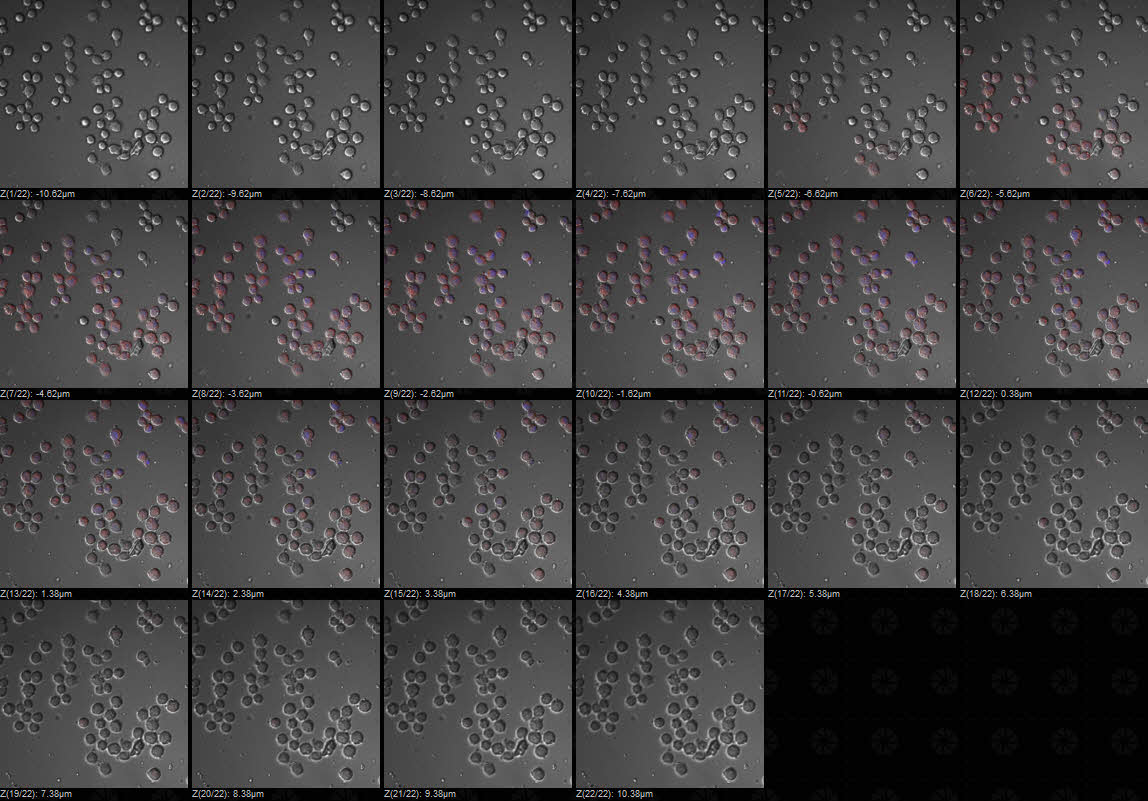


**S4 Fig. Rv2463 is internalized by J774 macrophages**. J774 cells were incubated with PE-streptavidin-biotin conjugated Rv2463 (25 μg/ml). Internalization of the antigen was monitored using confocal microscopy. Images show Z-stacks of 1μm section for Rv3416 at 30min post-stimulation.
