# Supplementary material for: Regulation of L-type Voltage Gated Calcium Channel CACNA1S in Macrophages upon Mycobacterium tuberculosis Infection
Source: PLoS One. 2015 Apr 27;10(4):e0124263. doi: 10.1371/journal.pone.0124263 (PMC4411123; doi:10.1371/journal.pone.0124263)
Supplement: S5 Fig — Mouse bone marrow derived macrophages were stimulated with Rv2463 at 25 μg/ml or infecected with 2 MOI M. tb H37Rv for 48h. CACNA1S expression on cell surface was monitored by flow cytometry. Bold lines represent stimulations with Rv2463 (left panel) or M. tb H37Rv (right panel) and thin lines represent unstimulated controls. Data from one of two independent experiments are shown. (DOC) [file pone.0124263.s005.doc]

**S5 Fig. Rv2463 and *M. tb* induce the upregulation of CACNA1S on bone marrow derived macrophages.** Mouse bone marrow derived macrophages were stimulated with Rv2463 at 25 μg/ml or infecected with 2 MOI *M. tb* H37Rv for 48h. CACNA1S expression on cell surface was monitored by flow cytometry. Bold lines represent stimulations with Rv2463 (left panel) or *M. tb* H37Rv (right panel) and thin lines represent unstimulated controls. Data from one of two independent experiments are shown.
